# The effect of intraperitoneal administration of BromAc on blood parameters: phase 1 study

**DOI:** 10.1007/s12672-021-00418-5

**Published:** 2021-08-16

**Authors:** Kevin Ke, Krishna Pillai, Ahmed H. Mekkawy, Javed Akhter, Samina Badar, Sarah J. Valle, David L. Morris

**Affiliations:** 1 Department of Surgery, St. George Hospital, Kogarah, NSW 2217 Australia; 2 Mucpharm Pty Ltd, Kogarah, NSW 2217, Australia; 3 St George & Sutherland Clinical School, University of New South Wales, Sydney, NSW 2217 Australia

**Keywords:** Pseudomyxoma peritonei, PMP, Bromelain, Acetylcysteine, BromAc®, Blood

## Abstract

Intraperitoneal administration of BromAc (bromelain + acetylcysteine) is currently undergoing a phase 1 clinical trial for pseudomyxoma peritonei at our institution. This study reports on analysis of routine blood parameters before and after treatment for a series of 25 patients in this trial. Blood parameters assessed included full blood count, electrolytes, urea, and creatinine, liver function tests, coagulation studies, as well as inflammatory markers (CRP). Certain parameters such as CRP, and white cell count, were significantly elevated after treatment whilst serum albumin level was reduced indicating an inflammatory reaction. However, liver enzymes, coagulation studies, and other parameters were not affected. Therefore, there are no additional safety signals evident upon analysis of routine blood parameter testing.

## Introduction

Pseudomyxoma peritonei (PMP) is a syndrome characterised by mucinous ascites, secreted by tumours of primarily appendix, ovary, or of bowel origin. It has an estimated incidence of 0.3 per 100,000 people, and a prevalence of 2 in 100,000 [1]. Current standard of care treatment involves cytoreductive surgery (CRS) with heated intraperitoneal chemotherapy (HIPEC), which has been reported to offer prolonged survival even when extensive disease is present. A multicentre registry incorporating data from 1548 patients with PMP undergoing CRS + HIPEC demonstrated 5-year survival of 58% [2]. However, this is a major procedure, with 33% of patients experiencing grade 3–5 morbidity [2]. Furthermore, treated patients may require repeat procedures owing to tumour recurrence [3, 4]. In a multicentre database of 1411 CRS + HIPEC procedures performed over two decades, it was reported that the rate of early recurrence in the first 5 years post-procedure was 25%. However, on closer inspection, the true figure is much higher, as it was reported that of the 262 patients followed up for over 10 years, 192 patients (75%) experienced a recurrence in the first 5 years, and only 33 patients (13%) had no recurrence after 10 years [5]. Repeat surgeries are more surgically complex, confer increased morbidity and can usually only be performed 2 or 3 times before patients are inoperable [5, 6]. Chemotherapy is largely ineffective due to tumor cells being engulfed by mucin, forming a protective barrier preventing chemotherapy penetration [7]. Clearly there is a need for improved therapies.

A therapy currently undergoing development at our institution is bromelain in combination with acetylcysteine (BromAc) for intraperitoneal therapy. Bromelain is derived from the stem of pineapple (*Ananas comosus*), containing a mixture of enzymes such as proteases, peroxidases, cellulases, glucosidases, esterases, glucosidases, etc. along with glycoproteins [8]. Acetylcysteine is an antioxidant commonly used for treating paracetamol toxicity [9] and as a mucolytic for treating cystic fibrosis [10] and chronic obstructive pulmonary disease (COPD) [11]. Bromelain is known to cleave peptide and glycosidic bonds while acetylcysteine reduces inter mucin disulphide linkages, resulting in the disintegration and solubilization of mucin when used in combination [12]. The safety and early feasibility of BromAc has been confirmed recently in a clinical trial where over 20 patients were treated successfully [13], although there was an elevation of C-reactive protein (CRP) in all patients indicative of inflammatory reaction that may been caused locally due to the proteolytic action of bromelain on tissues and blood vessels lining the peritoneal cavity. Further, since BromAc was delivered intraperitoneally to a highly vascular surface, it is envisaged that BromAc will gain entry systemically and hence may affect other organ systems. Therefore, we examined the levels of certain blood biomarkers, at baseline and post-treatment, to assess the effect of BromAc on organ systems.

## Methods

### General

The study involved patients with mucinous peritoneal tumour including PMP that were deemed inoperable. All patients were presented to a multi-disciplinary team (MDT) for suitability of trial entry and educated about the procedure prior to consenting. A pre-treatment computed tomography (CT) scan for assessment of the tumour as well as calculation of the size and the volume of tumour to be treated were required for discussion at the MDT meeting. Baseline blood tests including full blood count, liver function, renal function, C-reactive protein (CRP), fibrinogen and coagulation tests were also required. A Patient Information Booklet was given to the subjects for review and follow-up with any additional questions answered prior to consenting to treatment. Following completion of investigations and suitability for entry, if the patient was willing to provide informed consent, the form was signed. Educational and additional appointments with supporting services and further review with other disciplines were requested as required. The patient was provided with a signed copy of the patient information booklet, a fully executed copy of consent form and day of procedure instructions. A pre-treatment baseline symptom questionnaire and quality of life forms were completed.

On the day of the procedure, the patient was fasted for 6 h and administered oral antihistamine (Loratadine 10 mg) before treatment. Drug was administered directly into tumour or peritoneal cavity via drain and allowed to dwell for 24 h. Then, the tumour was drained, and repeat treatment was considered on subsequent days. Injection was performed under radiological guidance by an interventional radiologist through percutaneously inserted drain, remaining in situ for the treatment period. Aspiration and drainage were through this drain, and dosage was dependent on the calculated tumour dimensions and volume. Briefly, Bromelain (MucPharm Pty Ltd, Australia; Challenge Bioproducts Co Ltd, Taiwan) 20–45 mg (intra-tumoral injections) or 45–60 mg (intra-peritoneal injections) and Acetylcysteine (Acetylcysteine-Link, Link Pharma, Australia) 1–1.5 g (intra-tumoral injections) or 2 g (intra-peritoneal injections) were reconstituted and administered in 5% glucose through a sterile Millex 0.22 μm syringe filter. The injected volume injected, as feasible, was corresponding to 20% of the target tumour volume (intra-tumoral) or 500 mL (intra-peritoneal). The maximum daily dose was 60 mg Bromelain and 2 g Acetylcysteine. Further details regarding study design can be found in the referenced paper [13]. The trial is registered at Clinicaltrials.gov (NCT03976973) and anzctr.org.au (ACTRN12617001612303). Ethics approval for this study was provided by Bellberry Ltd (2017-07-561) and patients that did not meet criteria including retreatments were conducted via compassionate access using the same protocol and reporting requirements.

A total of 25 patients were treated with BromAc intraperitoneally from February 2018 to May 2020. There were in total 42 treatment periods recorded, with a treatment period defined as a set of treatments which were not separated by an interval of time longer than 7 days. Of these 42 periods, 32 were able to be analysed as blood parameters were recorded at suitable time points, from 20 different patients. Pre-treatment blood tests were taken any time from the period of 2 weeks prior to initiation of treatment, up until the day of treatment. Post-treatment blood tests were the first set of blood tests taken following the last treatment, taken within 1 week of their last dose.

### Analysis and quantification of routine blood biomarkers

All blood samples collected were analysed following standard laboratory protocol by the clinical pathology service of St George Hospital, Sydney (SEALS pathology).

### Blood bromelain quantification

Bromelain concentrations in plasma of patients were quantified using ELISA developed in house using pre coated 96 well plates with mouse monoclonal anti bromelain antibody at a dilution of 1/500 in coating buffer at 37 °C/24 h. The plates were then washed and treated to different concentrations ranging from 20 to 600 ng/mL of bromelain, incubated at 37 °C/2 h, washed with washing buffer and then treated with HRP labelled anti mouse antibody after which substrate was added following standard procedure [14].

### Statistical analysis

Results from the measurement of blood biomarkers were analysed using GraphPad Prism 9, and the statistical test used to assess for significance was the Wilcoxon matched-pairs signed rank test. *P*-values under 0.05 were judged to be significant.

## Results

### Patient and treatment parameters

The mean age of patients assessed was 64, with a range from 38 to 82 years old. Patients assessed received a mean dosage of 124 mg of bromelain, and 4.9 g of acetylcysteine per treatment period. Each treatment period consisted of a mean of 3.8 separate drug administrations over different days.

Additional subgroup analyses were performed, dividing the 32 treatment periods into 16 low dose periods (cumulative bromelain dose < 100 mg) and 16 high dose periods (cumulative bromelain > 100 mg). The low dose group received a mean of 64 mg of bromelain and 2.7 g of acetylcysteine. The high dose group received a mean of 183 mg of bromelain, and 7.2 g of acetylcysteine, approximately 3-times the dosage of the low dose group. Female patients as well as older patients were more likely to be treated with lower dosages compared to male patients and younger patients (Table 1). Dosing was calculated based on tumour volume. Patients were assessed after each injection for tolerability and response, and treatments were continued it was determined to be clinically beneficial and tolerable. The lowest dosage treatment period assessed consisted of 38 mg of bromelain and 1.9 g of acetylcysteine, while the highest dosage treatment period assessed was 325 mg of bromelain and 14 g of acetylcysteine.

**Table 1:** Patient parameters

	Low doses	High doses	All doses
Total treatment periods (n)	16	16	32
Mean age of all patients (years)	67	61	64
% Female	57	38	47
% Low dose (< 100 mg bromelain)	100	0	50
% High dose (> 100 mg bromelain)	0	100	50
Mean dosage of bromelain (mg)	65	183	124
Mean dosage of acetylcysteine (g)	2.7	7.2	4.9

### Blood biomarkers

**Fig. 1:**
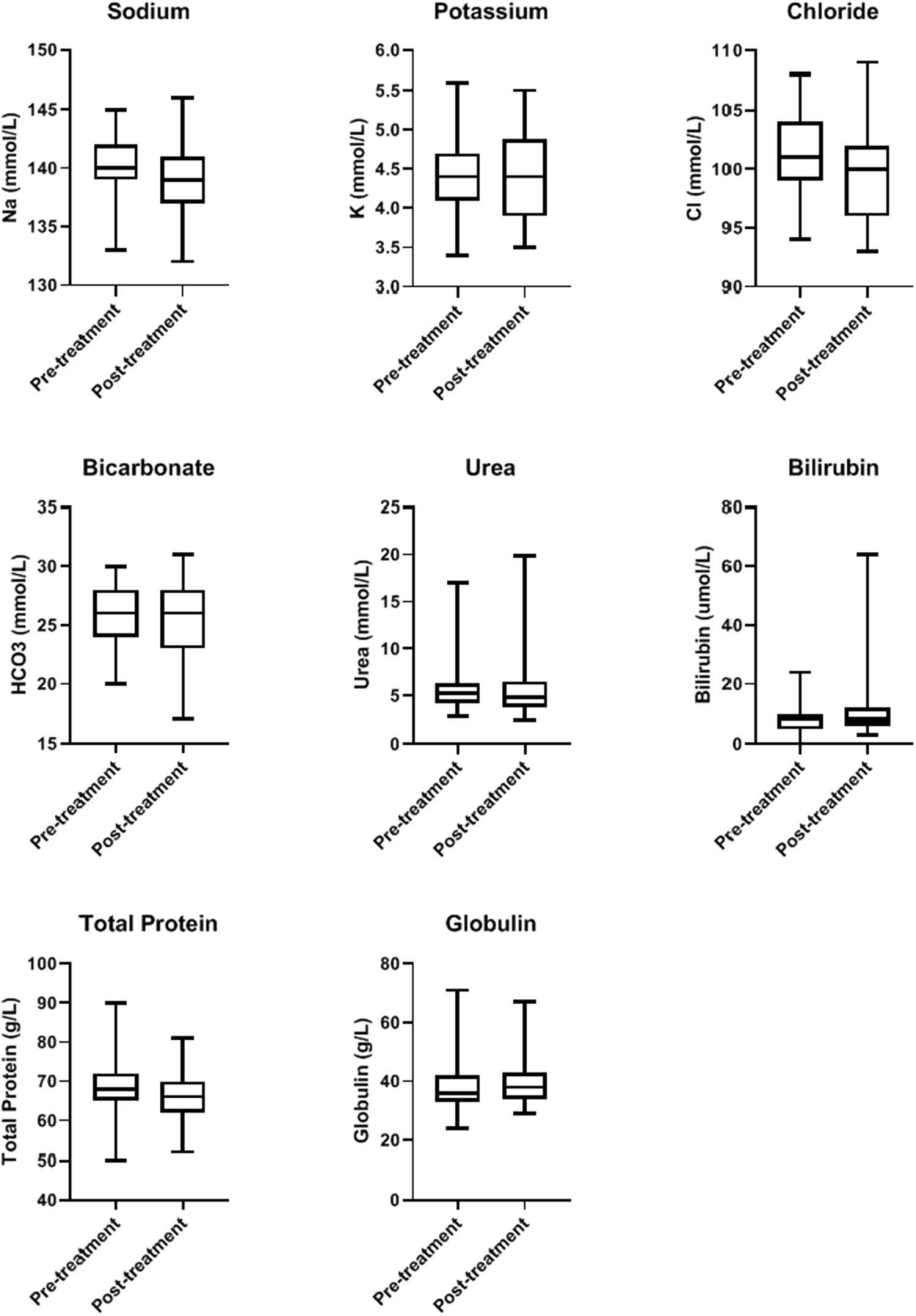
Shows the effect of BromAc treatment on blood level of sodium, potassium, chloride, bicarbonate, urea, bilirubin, total protein and globulin, before and after treatment in all patients. Sodium, chloride and total protein were slightly depressed post-treatment

**Fig. 2:**
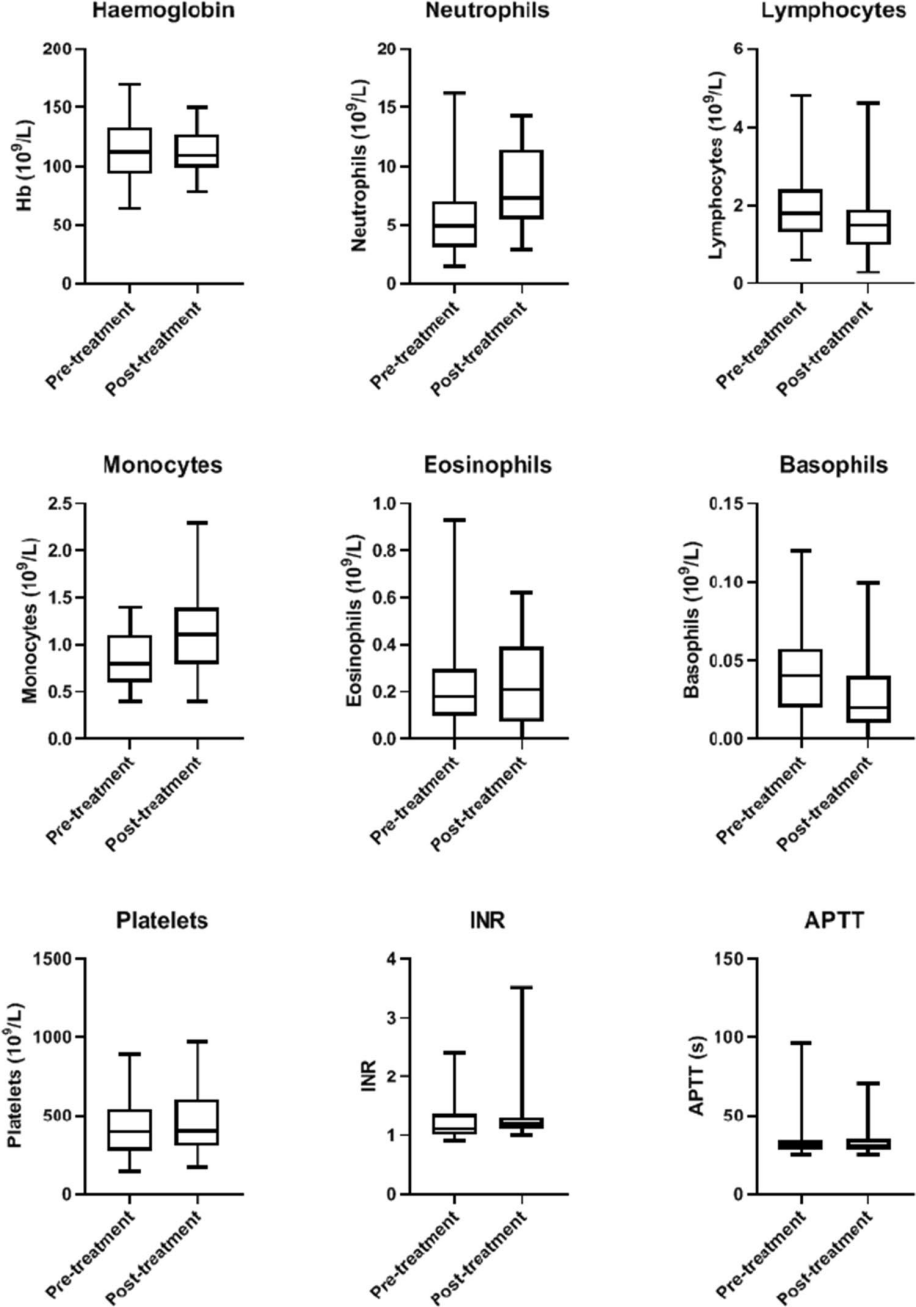
Shows the effect of BromAc treatment on blood level of haemoglobin, neutrophils, lymphocytes, monocytes, eosinophils, basophils, platelets, INR, and APTT, before and after treatment in all patients. *INR* international normalisation ratio, *APTT* activated partial thromboplastin time (s). Neutrophils, monocytes and eosinophils were slightly increased post-treatment whilst lymphocytes and basophils were slightly depressed

**Fig. 3:**
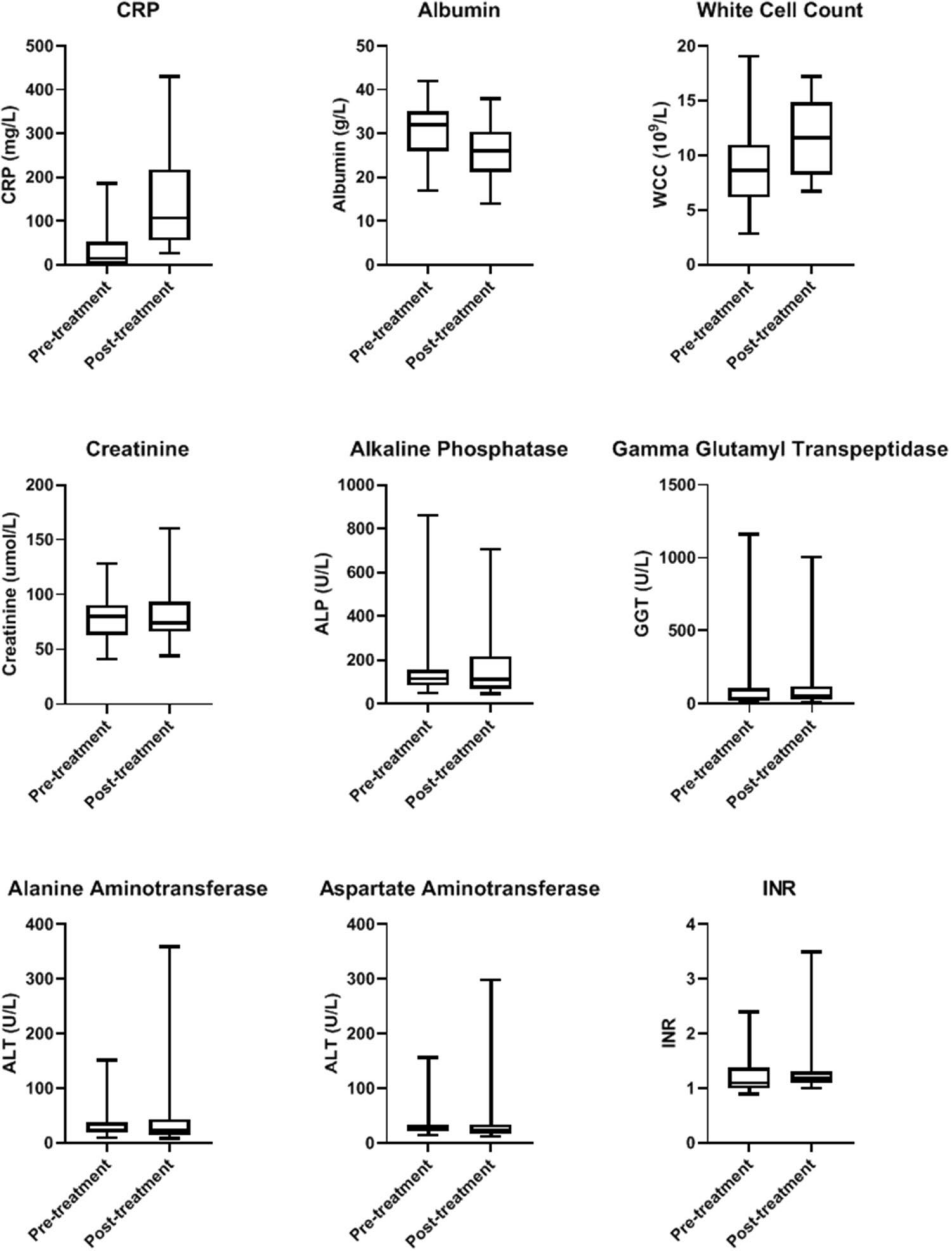
Shows the effect of BromAc treatment on blood level of CRP (C-reactive protein), albumin, white cell count, creatinine, alkaline phosphatase, gamma glutamyl transpeptidase, alanine aminotransferase, aspartate aminotransferase and INR before and after treatment in all patients. CRP and white cell count were increased whilst albumin and creatinine were slightly depressed post-treatment

Blood biomarker analysis and statistical evaluation of the whole treatment group indicated that various biomarkers were either impacted by treatment, evident when comparing their post-treatment to baseline blood parameter measurements (Table 2). In particular, CRP was substantially elevated from mean value of 43 to 142 mg/L post-treatment (p < 0.001). Other biomarkers that were enhanced are white blood cells (WCC), neutrophils and monocytes. Albumin and lymphocytes were on the other hand depressed (Table 2, Figs. 1, 2, and 3).

**Table 2:** Blood biomarkers in all BromAc treated patients

Parameter	Units	Baseline (mean ± SD)	Post-treatment (mean ± SD)	*p*-value	n
Sodium	mmol/L	140.03 ± 2.89	138.88 ± 3.19	0.07	32
Potassium	mmol/L	4.42 ± 0.55	4.41 ± 0.56	0.93	32
Chloride	mmol/L	101.42 ± 3.34	99.38 ± 3.78	0.002	31
Bicarbonate	mmol/L	31.31 ± 37.37	26.22 ± 2.61	0.6	31
Urea	mmol/L	5.9 ± 2.81	6.08 ± 3.79	0.63	31
Creatinine	µmol/L	77.16 ± 21.69	81.87 ± 25.61	0.36	31
Total bilirubin	µmol/L	8.34 ± 5.08	12.22 ± 13.98	0.31	32
Total protein	g/L	68.45 ± 9.01	65.94 ± 6.92	0.1	31
Albumin	g/L	30.25 ± 6.99	26.25 ± 5.85	0.01	32
CRP	mg/L	43.07 ± 56.58	142.42 ± 109.76	< 0.001	28
ALP	U/L	161.88 ± 159.6	176.19 ± 152.56	0.8	32
GGT	U/L	104.97 ± 210.06	112.75 ± 183.86	0.47	31
ALT	U/L	33.34 ± 28.73	40.44 ± 61.27	0.93	32
AST	U/L	32.55 ± 27.68	36.19 ± 51.91	0.51	30
Haemoglobin	10^9^/L	112.97 ± 24.91	112.09 ± 20.86	0.67	32
WCC	10^9^/L	9.06 ± 3.86	11.48 ± 3.36	0.001	32
Neutrophils	10^9^/L	5.9 ± 3.59	8.31 ± 3.15	0.002	31
Lymphocytes	10^9^/L	2.03 ± 1.02	1.62 ± 0.99	0.01	31
Monocytes	10^9^/L	0.86 ± 0.32	1.19 ± 0.5	0.002	31
Eosinophils	10^9^/L	0.23 ± 0.21	0.22 ± 0.17	0.73	31
Basophils	10^9^/L	0.04 ± 0.03	0.03 ± 0.02	0.03	31
Platelets	10^9^/L	426.28 ± 172.81	492.48 ± 238.39	0.2	32
INR	INR	1.24 ± 0.36	1.27 ± 0.45	0.73	28
APTT	s	34.76 ± 14.32	34.1 ± 9.39	0.6	29

*ALP* alkaline phosphatase, *ALT* alanine transaminase, *APTT* activated partial thromboplastin time, *AST* aspartate transaminase, *CRP* C-reactive protein, *GGT* gamma glutamyl transpeptidase, *INR* international normalized ratio, *SD* standard deviation, *WCC* white cell count

Both the low dosage and high dosage groups demonstrated sharp increases in CRP, and derangements to various white blood cell types (neutrophils, lymphocytes, monocytes). Both groups demonstrated numerical decreases in serum albumin concentration, although these were not statistically significant. There were no additional signals evident in any other blood parameters when conducting the subgroup analysis (Tables 3, 4, Figs. 1, 2, 3).

**Table 3:** Blood biomarkers in low dosage patients (< 100 mg bromelain)

Parameter	Units	Baseline (mean ± SD)	Post-treatment (mean ± SD)	*p*-value	n
Sodium	mmol/L	139.69 ± 3.03	138.5 ± 3.1	0.32	16
Potassium	mmol/L	4.42 ± 0.52	4.35 ± 0.61	0.81	16
Chloride	mmol/L	100.94 ± 3.7	99.25 ± 3.19	0.12	16
Bicarbonate	mmol/L	25.56 ± 3.5	25.56 ± 2.9	0.61	16
Urea	mmol/L	5.83 ± 3.35	6.18 ± 4.73	0.99	16
Creatinine	µmol/L	78.06 ± 20.52	82.81 ± 31.23	0.83	16
Total bilirubin	µmol/L	8.69 ± 5.69	13.13 ± 14.26	0.24	16
Total protein	g/L	68.56 ± 7.13	66.13 ± 6.9	0.26	16
Albumin	g/L	31.19 ± 6.81	28.19 ± 5.54	0.1	16
CRP	mg/L	37.6 ± 61.99	138.69 ± 118.76	< 0.001	15
ALP	U/L	169.13 ± 198.13	175.81 ± 184.16	0.75	16
GGT	U/L	134.38 ± 284.65	133.88 ± 252.5	0.65	16
ALT	U/L	41.69 ± 38.34	53.69 ± 84.1	0.89	16
AST	U/L	40.31 ± 36.94	43.75 ± 68.77	0.67	16
Haemoglobin	10^9^/L	118.75 ± 21.34	118.56 ± 19.5	0.95	16
WCC	10^9^/L	8.27 ± 3.66	11.45 ± 3.64	0.02	16
Neutrophils	10^9^/L	4.94 ± 3.33	8.19 ± 3.67	0.02	16
Lymphocytes	10^9^/L	2.19 ± 1.1	1.74 ± 1.19	0.05	16
Monocytes	10^9^/L	0.85 ± 0.3	1.26 ± 0.44	0.03	16
Eosinophils	10^9^/L	0.27 ± 0.24	0.24 ± 0.15	0.71	16
Basophils	10^9^/L	0.05 ± 0.03	0.03 ± 0.03	0.03	16
Platelets	10^9^/L	379.19 ± 146.2	390.44 ± 161.26	0.56	16
INR	INR	1.23 ± 0.37	1.34 ± 0.6	0.58	15
APTT	s	31.93 ± 5.7	32.8 ± 6.52	0.53	15

*ALP* alkaline phosphatase, *ALT* alanine transaminase, *APTT* activated partial thromboplastin time, *AST* aspartate transaminase, *CRP* C-reactive protein, *GGT* gamma glutamyl transpeptidase, *INR* international normalized ratio, *SD* standard deviation, *WCC* white cell count

**Table 4:** Blood biomarkers in high dosage patients (> 100 mg bromelain)

Parameter	Units	Baseline (mean ± SD)	Post-treatment (mean ± SD)	*p*-value	n
Sodium	mmol/L	140.38 ± 2.8	139.25 ± 3.34	0.21	16
Potassium	mmol/L	4.41 ± 0.59	4.48 ± 0.51	0.68	16
Chloride	mmol/L	101.93 ± 2.96	99.5 ± 4.4	0.004	15
Bicarbonate	mmol/L	37.06 ± 52.95	26.88 ± 2.19	0.27	16
Urea	mmol/L	5.99 ± 2.19	5.97 ± 2.72	0.54	15
Creatinine	µmol/L	76.25 ± 23.43	80.87 ± 18.93	0.24	15
Total bilirubin	µmol/L	8 ± 4.55	11.31 ± 14.1	0.85	16
Total protein	g/L	68.33 ± 10.93	65.75 ± 7.17	0.26	15
Albumin	g/L	29.31 ± 7.25	24.31 ± 5.67	0.06	16
CRP	mg/L	48.93 ± 51.81	146.4 ± 103.3	0.009	14
ALP	U/L	154.63 ± 115.22	176.56 ± 119.12	0.87	16
GGT	U/L	73.6 ± 75.45	91.63 ± 71.78	0.45	15
ALT	U/L	25 ± 9.36	27.19 ± 17.6	0.84	16
AST	U/L	24.27 ± 6.11	28.13 ± 23.94	0.75	15
Haemoglobin	10^9^/L	107.19 ± 27.49	105.63 ± 20.74	0.61	16
WCC	10^9^/L	9.84 ± 4.01	11.52 ± 3.17	0.02	16
Neutrophils	10^9^/L	6.85 ± 3.7	8.44 ± 2.62	0.05	15
Lymphocytes	10^9^/L	1.88 ± 0.95	1.49 ± 0.75	0.05	15
Monocytes	10^9^/L	0.88 ± 0.35	1.11 ± 0.57	0.05	15
Eosinophils	10^9^/L	0.19 ± 0.17	0.2 ± 0.19	0.99	14
Basophils	10^9^/L	0.04 ± 0.03	0.03 ± 0.02	0.6	14
Platelets	10^9^/L	473.38 ± 188.71	601.33 ± 263.29	0.25	15
INR	INR	1.26 ± 0.36	1.19 ± 0.13	0.63	14
APTT	s	37.79 ± 19.69	35.5 ± 11.84	0.92	14

*ALP* alkaline phosphatase, *ALT* alanine transaminase, *APTT* activated partial thromboplastin time, *AST* aspartate transaminase, *CRP* C-reactive protein, *GGT* gamma glutamyl transpeptidase, *INR* international normalized ratio, *SD* standard deviation, *WCC* white cell count

Of note, liver enzymes were not impacted by BromAc treatment in a statistically significant manner as previously thought, although numerically, the mean values of ALP, GGT, ALT and AST were slightly higher post-treatment (Tables 2, 3, 4). Platelets were not significantly impacted by treatment, nor were INR or APTT values (Table 5). There was also no increase in creatinine observed. In terms of serum electrolytes, there was a slight mean reduction of sodium concentration from 140 to 139 mmol/L, as well as a reduction in serum chloride concentrations from 101 to 99.5 mmol/L (p = 0.002) although these concentrations remained well within the normal reference ranges and are likely clinically irrelevant.

**Table 5:** Coagulation parameters across low and high dosages

	Parameter	Pre-treatment (mean ± SD)	Post-treatment (mean ± SD)	P-value
All patients	INR	1.2 ± 0.3	1.3 ± 0.4	0.73
	APTT (s)	35 ± 14	34 ± 9	0.60
Low dose	INR	1.2 ± 0.4	1.3 ± 0.6	0.58
	APTT (s)	32 ± 6	33 ± 6.5	0.53
High dose	INR	1.3 ± 0.3	1.2 ± 0.1	0.63
	APTT (s)	38 ± 19	36 ± 11	0.92

*APTT* activated partial thromboplastin time, *INR* international normalised ratio, *SD* standard deviation

BromAc delivered by intra-tumoral injection (IT) at 1.5 mg/mL resulted in a blood concentration of 110 ng/mL at 3 h, whilst intraperitoneal (IP) delivery at a concentration of 0.75 mg/mL resulted in blood concentration of 440 ng/mL at 2 h followed by a decline to 350 ng/mL at 5 h. Intraperitoneal delivery (0.75 mg/mL) may result in a higher absorption through peritoneal membrane as opposed to IT delivery at 1.5 mg/mL (Table 6).

**Table 6:** Blood serum level of bromelain at 2–5 h after initial treatment

Route	Drug administration				Blood bromelain concentration (ng/mL)		
	Brom (mg)	Ac (g)	Volume (mL)	Brom conc. (mg/mL)	2 h	3 h	5 h
IT	30	1.0	20	1.5	–	110	–
IP	30	1.5	40	0.75	440	–	350

*Brom* bromelain, *Ac* acetylcysteine, *volume* delivery volume, *IT* intra-tumoral administration, *IP* intraperitoneal administration

## Discussion

Importantly, the present investigation reveals no evidence of liver toxicity, kidney toxicity, or derangement to the coagulation profile of patients receiving the specified range of BromAc doses. In the initial publication of the clinical trial, it was speculated that drug administration was causing instances of raised liver enzyme serum concentrations [13]. However, this investigation raises the possibility that those reported cases may have purely represented statistically random fluctuations unrelated to BromAc administration. This is because AST, ALT, GGT, as well as ALP were not affected in a statistically significant manner in both the low and high dosage groups. Furthermore, although bromelain is known to have anticoagulative action owing to its biochemical interaction with the coagulation cascade [15], the dosage delivered in this investigation seems to have no effect upon coagulation related parameters. Therefore, this investigation forms an important part of our understanding of BromAc’s safety profile, supporting the case for further development of the drug as a novel mucolytic agent when given through the intraperitoneal or intra-tumoral route.

Analysis of the whole treatment group indicated that acute phase reactants such as CRP, white blood cell count (WCC), neutrophils and monocytes were increased in a statistically significant manner consistent with an acute phase reaction [16]. These changes were observed in both high dose and low dose subgroups consistently. Most noticeably, CRP was increased over twofold from the patients’ already elevated baseline concentrations. Other biomarkers were also enhanced significantly indicating inflammation, possibly at the site of BromAc delivery. The monocytes to lymphocytes ratio (MLR), neutrophil to lymphocytes ratio (NLR) and platelets to lymphocytes ratio (PLR) were all increased in a statistically significant manner indicating inflammation [17, 18]. The presence of high concentrations of acetylcysteine may also disrupt cellular surfaces, as it is well known that acetylcysteine enhances the absorption of certain antibiotics mainly through its reductive action on disulphide linkages found in cellular membrane proteins. Although bromelain is known for its anti-inflammatory properties [19], in the present investigation it has evoked an inflammatory reaction. The administration of intraperitoneal bromelain to mice has previously demonstrated disruption of peritoneal surfaces with mild bleeding and clotting reported [20, 21]. It is important to note that baseline values of neutrophils and platelets were higher in our patient population compared to the normal population, while lymphocytes were lower (Table 3) [22, 23]. This is likely due to a background level of chronic inflammation caused by late-stage cancer.

Albumin was consistently depressed in the present study, consistent with the presence of inflammation as it is a negative acute phase reactant [24]. Albumins are composed of different amino acids that are linked together by peptide bonds [25] whilst their molecular geometry is dictated by the disulphide bonds [26], hence they are highly prone to chemical reactivity by BromAc. This partial disintegration of albumin may contribute to the lower levels of albumin measured in blood, although this requires clarification in future studies. On the other hand, the generation of reactive radicals at the site of inflammation may attract albumin since it is an antioxidant that is capable of quenching harmful radicals [27]. Albumin is also known to enter extra-vascular spaces in order to attract fluid in order to initiate the healing process [28]. Albumin also may be sequestered in areas where there is an inflammatory reaction as a protective coat to prevent further degradation of cellular materials, and hence blood albumin may be down regulated [29].

In this investigation, we clearly observe an acute inflammatory process occurring following BromAc administration, supported by the presence of low-grade fever in about one third of patients [13]. Since bromelain consists of several enzymes such as proteases, peroxidases, phosphatases, esterases, glycosylases, cellulases etc., it is capable of exerting enzymic reaction on a variety of compounds including proteins and glycoproteins [30], thereby changing their molecular features. On the other hand, acetylcysteine exerts its action on disulphide bonds, and hence also changes the configuration of protein and other biological molecules containing disulphide bonds [31]. Individually, they have been shown to enhance the penetration of macromolecules and drugs through membranes and other cellular structures [8, 32]. Hence, owing to their chemical properties, the exposure of peritoneal surfaces to bromelain and acetylcysteine may initiate a chemical reaction affecting the cells lining the peritoneum, as well as blood vessels leading to inflammation. Beyond this, BromAc administration may also inadvertently release the components of cancer cells that are usually found intracellularly, by disrupting necrotic cell membranes and the surrounding mucinous barrier through drug action. The release of intracellular constituents into extracellular space is known to be a potent endogenous inducer of inflammation [33]. Furthermore, the large peritoneal surface may also absorb substantial quantities of bromelain and acetylcysteine potentially leading to systemic activity although the concentrations observed have yet to be demonstrated to have any effect. Future research into these areas is indicated.

We divided patients into two groups based on dosage (high and low dose) for post-hoc analysis, in order to explore if there were any potential blood marker effects that were impacted by the dosage given (see Table 3). We noticed a consistent picture between the two treatment groups, although there were numerical differences between groups which were difficult to interpret and inconsistent. Importantly, there could be a component of variation caused by the fact that high dose patients would have had a longer time elapse since their first dose of BromAc because of longer treatment time as they had more disease, confounding any interpretation of more acute parameters. Nevertheless, albumin appeared to be more significantly depressed in higher dosage patients potentially indicating a higher degree of inflammation. White blood cell and neutrophil concentration was increased by a proportionally higher amount in the low dose compared to the high dose group, although we note that the low dose group had a lower baseline to begin with because they are generally less ill patients (lower disease volume). Besides the identified differences in the length of treatment time, and drug dosage, differences in the low dose and the high dose groups may also be partly due to age differences, sex and other factors, in addition to their differences in the cancer stage. Most of the inflammatory markers are known to be affected, either down-regulated or up-regulated in cancer patients depending on the cancer stage [34]. Overall, we are unable to make any firm conclusions about dose related impacts of BromAc at the range of doses tested, as both high and low dose groups result in a reasonably consistent pattern of blood marker modulation when accounting for the variability of the dosages given.

We supplemented our investigation with an examination of blood bromelain concentrations in two patients, who had the same dosage of bromelain administered albeit with different delivery volumes and modes of delivery. Intraperitoneal delivery resulted in a much higher bromelain level of 440 ng/mL at 2 h as opposed to intra-tumoral delivery of 110 ng/mL at 3 h. Intra-tumoral delivery of bromelain relies on diffusion out of the mucinous tumour before penetrating the peritoneal membrane and it is slower compared to direct IP delivery where vast peritoneal surfaces are exposed to bromelain immediately. Although the concentration of bromelain in IT delivery was 1.5 mg/mL as opposed to IP of 0.75 mg/mL, the higher solution may have further enhanced absorption. Further the slightly higher acetylcysteine level of extra 0.5 g in IP delivery may have also influenced the penetration of bromelain. Bromelain has a high molecular weight (28,000 Daltons) and acetylcysteine may enhance the penetration of bromelain. Again, further study is required to clarify these preliminary hypotheses. Further pharmacokinetic analysis would be useful as it could support further development of BromAc administration through other routes of administration.

## Conclusion

Intraperitoneal treatment using BromAc resulted in blood parameter derangements suggestive of an acute inflammatory response. There were no significant derangements to any other blood parameters noted, including liver enzymes or coagulation parameters.

## Data Availability

The datasets generated during and/or analysed during the current study are available from the corresponding author on reasonable request.
